# Palatine tonsil metastatic colon adenocarcinoma. Case report

**DOI:** 10.1016/j.bjorl.2021.07.012

**Published:** 2021-10-18

**Authors:** Gabriela Morales Medina, Carmen Vallés Rodríguez, Isidora Rettig Infante, Manuela Bayona Romero, Elda Oyarzún Bahamonde, Ángela Enterría González

**Affiliations:** a Hospital Universitario Rio Hortega, Department of Otorhinolaryngology, Valladolid, Spain; b Hospital Universitario Rio Hortega, Department of Pathological Anatomy, Valladolid, Spain

## Introduction

Colorectal cancer is the third most common worldwide malignant disease. The 22% of colorectal cancer patients will have a remote metastasis at the time of diagnosis of the primary tumor and about half of them will develop one in the course of the disease, usually to the liver.^1^ There are few cases in which the distant metastasis involves the Palatine Tonsils (PT). In the English scientific literature, there were only 14 cases identified at the time of this article.^1^, ^2^ Metastatic tumors of PT account for less than 1% of tonsil malignancies and most of them have their primary tumor in skin, kidney, lung, stomach, and breast.^3^

## Case report

**Figure 1:**
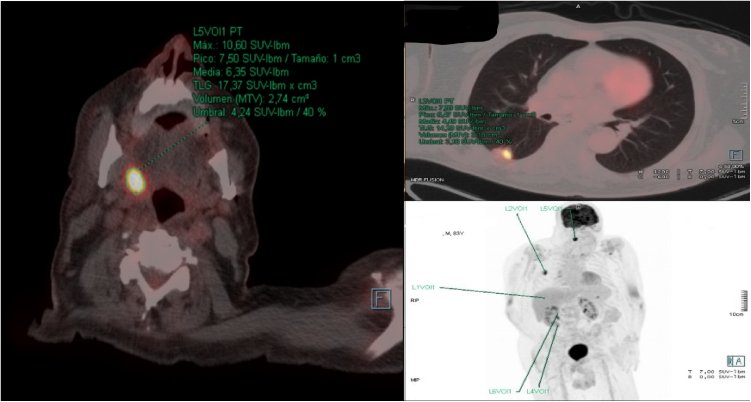
PET-CT showed an uptake of 18F-FDG in a nodule of the lower lobe of the right lung, and in the right palatine tonsil.

**Figure 2:**
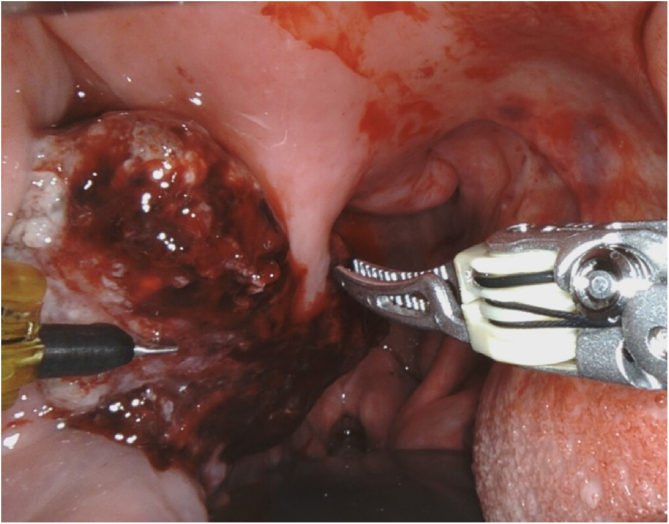
Friable exophytic mass at the right palatine tonsil with irregular borders and about 3 cm in diameter.

**Figure 3:**
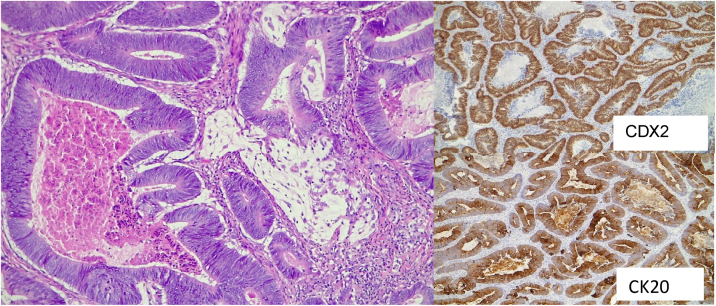
Histopathological examination of the resected palatine tonsil showed surface squamous epithelium with extensive infiltration of the tonsillar lamina propria by a glandiform epithelial neoplastic proliferation (H & E). The IHC study carried out in the neoplastic component showed positivity for CK20 and CDX2.

We report a case of an 83-year-old man with history of Lambda-type monoclonal IgG gammopathy, hypertension, carotid atherosclerosis, hypothyroidism, and Colon Adenocarcinoma (ADC) (pT3N0M0) Stage II, according to the American joint Committee on Cancer.^4^ The patient underwent right hemicolectomy in 2018 and no adjuvant treatment was required. After two years of being regularly followed up, a thoracic-abdominal Computed Tomography (CT) revealed a suspicious pulmonary nodule. Positron Emission Tomography scan (PET-CT) was performed, and it showed an uptake of [18F] fluoro-2-deoxy-D-glucose (18F-FDG) in the nodule of the lower lobe of the right lung, and in the right PT (Fig. 1). The patient presented odynophagia and mild dysphagia. Neither hoarseness nor dyspnea was referred. Physical examination showed a friable exophytic mass at the right PT with irregular borders and about 3 cm in diameter (Fig. 2); the rest of the physical exam was unremarkable. Incisional biopsy of the mass confirmed MT of colon ADC, being positive for Cytokeratine 20 (CK20), intestinal specific transcription factor (CDX2) and vilin in immunohistochemistry (Fig. 3). A core needle biopsy of the lung lesion was positive for colon ADC metastasis. After discussing the case in a multidisciplinary group, the systemic treatment with chemotherapy was rejected, and decided to remove the tonsil tumor and treat the lung metastasis with stereotactic radiotherapy. The patient underwent right radical tonsillectomy by Transoral approach with Robotic Surgery (TORS). Even though the surgery went without any incidents, during the third postoperative day the patient presented with acute confusional state and suddenly died, without knowing the immediate cause. The family refused the necropsy.

## Discussion

The PT are one of the most common sites of head and neck squamous cell carcinomas in adults, as well as lymphomas in children.^5^ Only 0.8% of the malignant tumors in this location are metastases.^2^ The most accepted route of metastatic spread is the hematogenous, through the portal vein or through the paravertebral plexus to the arterial circulation system of the PT.^1^ Retrograde lymphatic pathway has also been proposed, due to PT do not have afferent lymphatic vessels, being this last theory the least accepted.^5^ In the gastrointestinal tract, the tumors that most frequently metastasize to the PT are the gastric tumors, followed by the rectum and colon. In cases of PT metastasis from colorectal cancer, the mean age of diagnosis is 54 years and affects men more often^5^; they are usually unilateral, with the left side being the most affected.^3^ The last two characteristics differ from our case. Despite the low frequency of these lesions, it should be considered in the differential diagnosis of palatine tonsil`s lesions. Sometimes, even though the advances in imaging and molecular testing, identification of the primary is challenging.^3^ Immunohistochemical characteristics such as positivity for CK20, CDX2 and beta catenin as well as villin are useful complements to confirm the colorectal origin.^2^, ^5^ Immunohistochemical analysis continues to be the main choice to identify the histological origin of tonsil tumor with unknown primary.^3^ Colorectal cancer metastases in PT are generally considered a systemic disease with a poor prognosis^5^ and management depend on the characteristics of each case, demanding a multidisciplinary approach.

## Conclusion

Although rare, metastases to palatine tonsils should be considered if we are dealing with a patient with a tonsil lesion and a history of colorectal cancer. A clinical-pathological relationship and an immunohistochemical study associated with complementary tests must be established to reach the diagnosis. The prognosis of these patients is poor, so management must be carried out in a multidisciplinary approach. This case recalls the importance of ruling out distant metastases in unusual sites.

## Conflicts of interest

The authors declare no conflicts of interest.
